# A 20-YEAR COHORT STUDY OF LIVING ARRANGEMENTS AND COGNITIVE DECLINE IN THE MEXICAN AMERICAN POPULATION

**DOI:** 10.1093/geroni/igz038.2614

**Published:** 2019-11-08

**Authors:** Phillip A Cantu

**Affiliations:** University of Texas at Austin, Austin, Texas, United States

## Abstract

Backgrounds/Objectives: The prevalence of dementia in the elderly Latino population is expected to significantly increase from around 200,000 cases in 2000 to as many as 1.3 million cases in 2050. This demographic trend has important consequences for options in care and living arrangements. Very little is known about how of cognitive impairment trajectories co-vary with support available to Mexican Americans. We examine the relationship between living arrangements and the social support of individuals with dementia. Methods: Using data from nine waves from the 23 years of the Hispanic EPESE (n=3,952), we examine trajectories of cognitive functioning and family and social support. We first describe the change in Mini Mental Status Examination (MMSE) scores for survivors from Wave 1 (1993/1994) to Wave 9 (2015/2016), n=255. Growth Mixture Modeling (GMM) is then used to assess how changes in MMSE scores are distributed among living arrangements for individuals living independently compared to household extension (living with others) using the full sample. Results: Analyses reveal different trajectories in MMSE score. 12% (n=27) of the sample had no decrease, while the remaining (88%) were split between moderate decline (60% n=136, 1-10 point decline in MMSE) and severe decline (28% n=62 >10 point decline In MMSE). Changes in living arrangement over the same period show that 89% of individuals who move from independent living into extended household experienced cognitive decline. Conclusions: This study provides new information on how cognitive trajectories are associated with living arrangements. We discuss implications for improving community-based interventions for Latino family caregivers.

